# Functionalization of 3D Printed Polylactic Acid by Supercritical CO_2_ Impregnation with Mango Leaf Extract and Evaluation with Endothelial Colony-Forming Cells and Mesenchymal Stromal Cells

**DOI:** 10.3390/antiox15040454

**Published:** 2026-04-04

**Authors:** Ismael Sánchez-Gomar, Mercedes Cáceres-Medina, Cristina Cejudo-Bastante, Casimiro Mantell-Serrano, Lourdes Casas-Cardoso, Mª Carmen Durán-Ruiz

**Affiliations:** 1 Department of Biomedicine, Biotechnology and Public Health, Faculty of Medicine, University of Cádiz, 11003 Cádiz, Spain; caceresmedinamercedes09@gmail.com; 2 Biomedical Research and Innovation Institute of Cadiz (INiBICA), 11002 Cádiz, Spain; 3 Chemical Engineering and Food Technology Department, Faculty of Science, Wine and Agrifood Research Institute (IVAGRO), University of Cádiz, 11510 Puerto Real, Spain; cristina.cejudo@gm.uca.es (C.C.-B.); casimiro.mantell@uca.es (C.M.-S.); lourdes.casas@uca.es (L.C.-C.)

**Keywords:** PLA, supercritical carbon dioxide, supercritical impregnation, 3D-printing, mango leaf extract, polyphenols, DPPH, AAI, ECFCs, MSCs

## Abstract

Poly(lactic acid) (PLA) devices can be functionalized with plant-derived bioactives to introduce antioxidant activity while maintaining manufacturability and cytocompatibility. Here, a polyphenol-rich mango leaf extract (MLE) was obtained by enhanced solvent extraction and incorporated into PLA using supercritical carbon dioxide-assisted impregnation. Two manufacturing sequences were compared: impregnation after three-dimensional (3D) printing of discs and impregnation of filaments prior to printing. Extract yield and radical scavenging capacity were quantified, and impregnation efficiency was assessed as a function of pressure and temperature. Biological performance was evaluated using adipose tissue-derived endothelial colony-forming cells (ECFCs) and adipose tissue-derived mesenchymal stromal cells (MSCs), cultured separately and in co-culture on functionalized substrates. Impregnation after printing provided higher and more reproducible loading while preserving disc geometry, whereas impregnation before printing promoted swelling and printing-associated deformation that compromised structural fidelity. Cell-based analyses supported improved adhesion, spatial distribution, and proliferative status on discs produced by impregnation after printing under low-temperature and high-pressure conditions, without evidence of selective loss of either population in co-culture by flow cytometry. These results support post-print supercritical impregnation as a robust route to generate antioxidant, cell-supportive PLA scaffolds from agricultural by-products with potential relevance for vascular-oriented biomedical applications.

## 1. Introduction

Biodegradable polymers have emerged as revolutionary materials in the biomedical sector, offering innovative solutions for a wide range of applications. Unlike traditional polymers, these materials are designed to degrade in a controlled manner in the biological environment, transforming into biocompatible, non-toxic products that can be metabolized or excreted by the body [1]. This key feature eliminates the need for a second surgical procedure to remove the implant or device, reducing patient trauma and healthcare costs [2].

The most prominent biodegradable polymers in biomedicine include poly(lactic acid) (PLA), used in sutures [3], bone implants [4], stents [5] and drug delivery [6]; poly(glycolic acid) (PGA), mainly for sutures [7]; and its copolymer poly(lactide-co-glycolide) (PLGA), widely used in drug delivery systems [8] and tissue engineering [9]. Polycaprolactone (PCL) is used for long-term applications such as implants and tissue scaffolds [10,11,12,13]. Polydioxanone (PDO) is widely used in sutures and surgical staples [14,15]. Polyhydroxyalkanoates (PHAs) are being investigated for cardiovascular applications and nerve repair [16,17]. Finally, modified natural polymers such as chitosan and collagen are used in wound dressings and tissue engineering [18]. The aim is to provide biocompatible solutions that are safely degraded in the body.

The incorporation of pharmacoactive substances into polymeric biomedical devices may improve their biological integration after implantation. In this context, drug impregnation of polymeric matrices represents an important strategy for the development of more effective therapies with fewer adverse effects, as it enables controlled and sustained release of the active compound at the target site. Likewise, the incorporation of natural bioactive compounds into polymer-based medical devices may further enhance the functional performance and host response of these implanted systems. Several strategies have been employed to fabricate drug-eluting polymeric implants and to modulate the release of the incorporated active agents, including hot-melt extrusion [19], solvent casting, surface coating of preformed implants with the bioactive compound [20] and immersion of the implant in a solution containing the active molecule [21]. Nevertheless, these conventional approaches often present important limitations, such as the use of elevated processing temperatures that may compromise thermolabile compounds, or the reliance on organic solvents that require extensive removal and purification steps to comply with FDA regulations. Accordingly, alternative processing strategies have been explored to address these limitations. Among them, supercritical fluids offer important advantages over conventional techniques due to their physicochemical properties, which combine features of both liquids and gases, together with their negligible surface tension. In particular, supercritical CO_2_ (scCO_2_), with a critical temperature of 31.1 °C and a critical pressure of 73.8 bar, is a non-toxic and non-flammable solvent that enables the production of formulations and materials without leaving harmful solvent residues [22,23].

scCO_2_-assisted impregnation has been used to incorporate drugs such as cefazolin into bone chips to prevent infection in orthopedic procedures [24], ibuprofen into gelatin–siloxane cryogels suitable for controlled drug release and tissue engineering [25], and ketoprofen into mesoporous silica [26] and moxifloxacin in contact lenses to treat keratitis [27], among others. Another application of scCO_2_ impregnation is the incorporation of bioactive natural extracts into polymers used in biomedicine. Previous studies investigated the impregnation of olive leaf extract into biomedical-grade thermoplastic polyurethane (TPU) and PLA filaments under pressures ranging from 100 to 400 bar and temperatures between 35 and 55 °C. Under the most favorable operating conditions, TPU exhibited an extract loading and antioxidant activity approximately fourfold higher than those obtained for PLA [28]. The use of winemaking by-products to functionalize PLA for biomedical applications has also been successfully studied. For impregnated filaments, the impregnation load was particularly dependent on the extract, providing up to 8% (extract mass/polymer mass) impregnation [29]. In addition, extracts obtained from (*Mangifera indica* L.) mango leaves (MELs) are a source of bioactive compounds with recognized biological activity. One of the most abundant compounds isolated from their leaves is mangiferin, a metabolite that has been shown to present anti-inflammatory and antioxidant properties in several studies [30]. In addition, mangiferin has also been shown to reduce the apoptosis of cardiomyocytes during heart failure [31]. A previous study examined the potential of natural antioxidants extracted from mango leaves on the therapeutic capacity of endothelial colony-forming cells (ECFCs), which are considered promising for revascularization of ischemic areas in atherosclerotic patients in the treatment of cardiovascular diseases. The findings indicated that low concentrations of MLE improved angiogenic capacities of ECFCs and reduced proliferation, apoptosis, and the inflammatory response of these cells. Therefore, mango leaf extract was selected not merely as a generic antioxidant-rich plant source, but because previous evidence supported its cytocompatibility and its beneficial effects on vascular-related cells, which are directly relevant to the aims of the present study [32]. The scCO_2_-assisted impregnation of PLA with MEL was also investigated. The results confirm that PLA is an excellent material for delivering anti-denaturing and antioxidant compounds to the patient’s body, although further in vitro and in vivo studies are required [33]. In this regard, Grosso et al. [34], evaluated supercritical fluid impregnation in two different configurations: (a) PLA filaments impregnated with MEL and then used in 3D printing (SCI + 3D) and (b) 3D printed discs with PLA filaments that were then impregnated with scCO_2_ (3D + SCI). The best results in terms of cell viability of ECFCs were obtained with the 3D + SCI configuration impregnated at low temperature (35 °C). The cells exhibited an elongated polygonal morphology, characteristic of healthy proliferating endothelial cells. This morphology suggests that the extract provides an optimal environment for growth and maintenance of the culture. Taken together, these previous biological and technological findings make MEL a particularly suitable candidate for the development of bioactive PLA-based platforms [32,33].

In this study, we aimed to evaluate the feasibility and biological performance of mango leaf extract functionalized PLAs produced by combining scCO_2_ impregnation with 3D-printing. First, mango leaf extract was obtained by enhanced solvent extraction and characterized in terms of yield and antioxidant capacity using the 2,2-diphenyl-1-picrylhydrazyl (DPPH) assay and the antioxidant activity index. Second, the extract was incorporated into PLA by comparing two manufacturing sequences: impregnation after printing of discs and impregnation of filaments prior to printing, under defined pressure and temperature conditions, and loading was quantified gravimetrically. Third, we assessed the ability of the functionalized materials to support ECFCs and mesenchymal stromal cells (MSCs) in monoculture and in co-culture using quantitative fluorescence microscopy, flow cytometry-based discrimination of cell populations, and proliferation and apoptosis readouts. This integrated design provides a direct comparison of manufacturing sequences and operating conditions, enabling the identification of process parameters that maximize loading while preserving print fidelity and cellular compatibility, thereby contributing to the valorization of mango leaf by-products through a green processing strategy.

## 2. Materials and Methods

### 2.1. Raw Material and Chemicals

*Mangifera indica* L., Kent variety, leaves were provided by the “Estación Experimental La Mayora”, Centro Superior de Investigaciones Científicas (CSIC), Málaga, Spain. The leaves were dried at room temperature to constant weight and stored in the absence of light. For the impregnation process, 1.75 mm diameter polylactic acid (PLA) filaments were purchased from Mundo Reader S.L. (Madrid, Spain).

For extraction and impregnation operations, carbon dioxide (99.99%) was supplied by Carburos Metálicos, S.A. (Sant Esteve Sesrovires, Spain), and ethanol (96.49%) was provided by Alcoholes del Sur, S.A. (Córdoba, Spain). 2,2-Diphenyl-1-picrylhydrazyl (DPPH), purchased from Sigma-Aldrich (Steinheim, Germany), was used to evaluate antioxidant capacity. HPLC-grade formic acid (98%) and acetonitrile were supplied by Panreac Química S.L.U. (PanReac AppliChem ITW Reagents; Barcelona, Spain).

### 2.2. Enhance Solvent Extraction (ESE)

**Figure 1:**
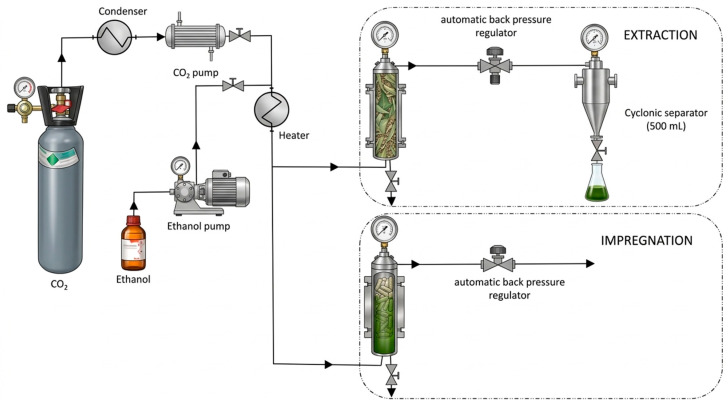
Schematic representation of the supercritical fluid system used for extraction and impregnation processes. Liquid CO_2_ from the cylinder was condensed and delivered by a CO_2_ pump, while ethanol was introduced as co-solvent by a separate pump. After heating, the pressurized mixture was directed either to the extraction module, containing the packed extraction vessel and a 500 mL cyclonic separator with automatic back-pressure regulation, or to the impregnation module, also equipped with automatic back-pressure regulation. The diagram highlights the main components and flow path of CO_2_ and ethanol throughout the system.

Extractions were performed using a Thar Technologies SF1000 system (Pittsburgh, PA, USA), equipped with a 1 L extractor and two high-pressure pumps (max. flow rate of 50 g/min each) for the independent delivery of CO_2_ and ethanol. Process parameters were precisely managed via an automatic back-pressure regulator and a thermostatic jacket to maintain thermal stability. The system layout is illustrated in Figure 1.

For the sample preparation, mango leaves were crushed to a particle size of 2–3 mm. Then, 200 g of the plant material was loaded into a paper cartridge and placed within the high-pressure vessel, which contained 500 mL of ethanol. Ethanol was selected as the co-solvent due to its eco-friendly nature, low toxicity, and proven efficiency in recovering polyphenolic compounds. The extraction was initiated by pressurizing the system with CO_2_ at a constant flow of 10 g/min until reaching 200 bar. Following the methodology described in previous studies [34], the process was conducted in batch mode at 80 °C for 24 h. Upon completion, the system was depressurized, and the resulting extract was collected and stored at 4 °C in the dark to ensure stability. All procedures were performed in duplicate to ensure reproducibility. All extraction experiments were performed in duplicate (*n* = 2).

### 2.3. Characterization of the Extracts

The extract was characterized in terms of total extraction yield, antioxidant activity, and the quantity of phenolic compounds. The extraction yield (%Y) was calculated as the ratio of the total dry extract mass (md) divided by the initial mass of the leaves (mi) according to Equation (1):

(1) $$\%Y=\frac{m_{d}}{m_{i}}*100$$

The extraction yields were determined in duplicate.

The antioxidant capacity of the extract was assessed using the DPPH (2,2-diphenyl-1-picrylhydrazyl) free radical scavenging assay [35]. DPPH is a stable free radical characterized by an unpaired electron, which can be neutralized by antioxidant compounds through hydrogen atom donation. This reduction is accompanied by a color change from purple to yellow and a corresponding decrease in absorbance, which was monitored spectrophotometrically at 515 nm.

For the assay, 293 μL of a 60 μM ethanolic DPPH solution was mixed with 7 μL of extract aliquots prepared at different concentrations (48–2400 μg/mL) in ethanol. After incubation for 2 h at room temperature in the dark, absorbance was recorded at 515 nm using a Cary 60 UV–Vis spectrophotometer (Agilent Technologies, Santa Clara, CA, USA). A control was prepared by replacing the extract aliquot with 7 μL of ethanol. The percentage of oxidation inhibition (%I) was calculated by comparing the absorbance of the control (A0) with that measured after 2 h (Ai), according to Equation (2):

(2) $$\%\;I=\;\frac{\left(A_{0}-A_{i\;}\right)}{A_{0}}\;*100$$

The half-maximal inhibitory concentration (IC_50_), defined as the extract concentration required to inhibit oxidation by 50%, was graphically determined by plotting the percentage inhibition (%I) against the extract concentration. Lower IC_50_ values indicate higher antioxidant capacity.

The results are also expressed as the antioxidant activity index (AAI). According to Scherer and Godoy [36] this parameter is defined as the ratio between the initial DPPH concentration in the reaction medium and the half-maximal inhibitory concentration (IC50) (Equation (3)). The assay was performed in duplicate, and the standard deviation was calculated.

(3) $$\mathrm{AAI}=\frac{\left[\mathrm{DPPH}\right]\;(\frac{\mu g}{\mathrm{mL}})}{{\mathrm{IC}}_{50\;}(\frac{\mu g}{\mathrm{mL}})}$$

The main phenolic compounds in MEL were quantified by HPLC as previously described [34], using a Jasco Corporation system (Madrid, Spain). Separation was achieved on a Fortis C18 column (150 mm × 3 mm, 5 μm; Fortis Technologies Ltd., Neston, Cheshire, UK), also supplied by Jasco Corporation. The mobile phase was composed of 0.1% formic acid in water (solvent A) and 0.1% formic acid in acetonitrile (solvent B). A sample volume of 20 μL was injected in all cases. Elution was carried out at a flow rate of 1 mL/min using the following linear gradient program [t (min), %B]: (0, 0), (0.2, 0), (0.3, 7), (14.7, 8.5), (40, 19), (45, 33), (48, 50), (50, 95), (57, 0), and (63, 0). Chromatographic data were acquired and processed using Jasco ChromNAV software (version 2.04.04). All extract characterization assays, including DPPH analysis and HPLC quantification, were performed in duplicate (*n* = 2).

### 2.4. 3D Printing

Discs were printed using Ultimaker Cura 5.10 computer software and an ANYCUBIC Mega S 3D printer. The printing temperature was 200 °C and the printing rate was 25 mm/s. Two different configurations were evaluated using the methodology proposed by Grosso et al. [34]. The first configuration involves printing PLA discs and then impregnating them with scCO_2_ in the presence of MLE as the active extract (3D + SCI). The second configuration involves impregnating a PLA filament with MLE, followed by printing discs with this filament. This configuration is called SCI + 3D.

### 2.5. Impregnation at High Pressure (SCI)

Impregnation of MLE into PLA using scCO_2_ was carried out using high-pressure equipment from Thar Technologies (Pittsburgh, PA, USA), similar to that used in the extraction process (Figure 1). The SF100 model, featuring a 100 mL vessel, was used to impregnate the 3D + SCI discs. In contrast, the SF1000 model (1 L capacity) was used for the SCI + 3D discs, which were impregnated in their original filament conformation prior to printing. The primary difference between the setups was the volume of the vessel and the amount of initial PLA added; other components, such as pumps and heat exchangers, were similar.

For the impregnation, the extract was added to the bottom of the vessel at a volume corresponding to 3% of the reactor’s capacity. To prevent direct contact between the polymer and the extract, the PLA filaments or discs were placed in a steel support basket. The process was conducted in batch mode: CO_2_ was first pumped at a flow rate of 10 g/min until reaching the target pressure. The flow was then stopped, and the pressure was maintained throughout the impregnation period. Finally, the system was rapidly depressurized (40 bar/min) to facilitate impregnation of the PLA.

Any excess extract was removed from the filaments using a damp lint-free cloth (o moist tissue). Experiments were performed in duplicate, and the impregnated samples were stored in the dark to prevent degradation.

To evaluate the influence of processing conditions, two pressure levels (100 and 400 bar) and two temperatures (35 and 55 °C) were tested, covering a wide range of CO_2_ densities. At 35 °C, the CO_2_ density values were 700.1 and 972.2 kg/m^3^ at pressures of 100 and 400 bar, respectively. At 55 °C, the corresponding values were 337.2 and 906.8 kg/m^3^. The experiments were conducted for 1 h. All impregnation experiments were performed in duplicate (*n* = 2).

### 2.6. Characterization of the Impregnated Polymers

To determine the MLE loading on the impregnated polymer, the samples were chemically dissolved in dichloromethane (CH_2_Cl_2_). The absorbance of the resulting solutions was then quantified using a UV–VIS spectrophotometer (UV Mini-1240, Shimadzu, Kyoto, Japan).

A calibration curve was initially established using extract concentrations ranging from 32 to 900 mg/mL in 3 mL of CH_2_Cl_2_. To account for potential matrix effects, a fixed amount of neat polymer (60 mg) was added to each standard solution, ensuring that the polymer’s presence did not interfere with the readings. The measurements were performed at 660 nm, as this wavelength corresponds to the extract’s maximum absorbance while minimizing interference from other components in the medium.

The final concentration was calculated from the corresponding calibration curve in the same solvent (Equation (4)):

(4) $$y=0.0017*C-0.057\;\;\;\;\;\;\;\;\;\;\;\;\;\;R^{2}=0.9961$$

where C is the MEL concentration, mg/mL. Finally, the loading percentage (% L) was calculated from Equation (5):

(5) $$\%L=\frac{m_{MLE}}{m_{POL}}*100$$

where mMLE is the mass of extract impregnated (g) and mPOL is the mass of the polymer (g) employed in each assay. All experiments were performed in duplicate (*n* = 2).

To examine how the impregnation process affected PLA surface features, samples were prepared for SEM analysis after being reduced to smaller fragments. Prior to observation, each fragment was covered with a 10 nm conductive gold coating using a Cressington 208 HR device (Cressington Scientific Instruments, Watford, UK). Imaging was subsequently performed with a Nova NanoSEM 450 system (FEI, Hillsboro, OR, USA).

### 2.7. Isolation and Culture of ECFCs and MSCs

Both ECFCs and MSCs were obtained from white adipose tissue, as previously described by Lin et al. [37]. Briefly, ECFCs were seeded on plates coated with 1% gelatin (Sigma-Aldrich, St. Louis, MO, USA) and cultured in EGM-2 basal medium (Lonza, Basel, Switzerland), excluding hydrocortisone, supplemented with 20% fetal bovine serum (Gibco, Thermo Fisher Scientific, Waltham, MA, USA) and 1× glutamine–penicillin–streptomycin (Gibco, Thermo Fisher Scientific, Waltham, MA, USA). MSCs were cultured in MSC Growth Medium 2 (PromoCell, Heidelberg, Germany) supplemented with 10% FBS (Gibco, Thermo Fisher Scientific, Waltham, MA, USA) and 1× GPS (Gibco, Thermo Fisher Scientific, Waltham, MA, USA). Cell characterization was performed as previously described by Sánchez-Gomar et al. [32]. All cell populations were used between passages 5 and 7.

### 2.8. Cell Viability Assay

To evaluate the viability of ECFCs and MSCs cultured on PLA discs impregnated with mango leaf extract (MLE), 50,000 cells of each cell type were seeded onto the discs and maintained for 72 h in EBM-2 complete medium (Lonza, Basel, Switzerland). Cell adhesion and spatial distribution were assessed by Calcein staining (Calcein, Sigma-Aldrich, St. Louis, MO, USA) and quantified by fluorescence microscopy. Calcein-positive cells were counted and normalized to surface area (cells per mm^2^) using image-based analysis. Based on previous results from our group [34], cell assays were performed using the disc conditions described above, in triplicate (*n* = 3). Quantification of adherent cells was performed using Fiji (Fiji Is Just ImageJ, version 2.14.0/1.54f; National Institutes of Health, Bethesda, MD, USA), enabling the identification of the impregnation condition that most effectively supported the growth of each cell type.

### 2.9. Flow Cytometry Based Quantification of ECFCs and MSCs

To distinguish ECFCs from MSCs adherent to PLA discs, flow cytometry analysis was performed using CD31 as an endothelial-associated surface marker (anti-human CD31 antibody, BioLegend, San Diego, CA, USA; Cat. 303103). Briefly, 50,000 ECFCs and 50,000 MSCs were co-seeded onto PLA discs corresponding to conditions 1 and 2 and incubated for 72 h in EGM-2 complete medium (Lonza, Basel, Switzerland) supplemented with 20% FBS (Gibco, Thermo Fisher Scientific, Waltham, MA, USA) and 1% glutamine–penicillin–streptomycin (Gibco, Thermo Fisher Scientific, Waltham, MA, USA). After incubation, cells were detached from the discs using trypsin (Gibco, Thermo Fisher Scientific, Waltham, MA, USA) and stained with anti-CD31 antibody for 30 min. Samples were acquired on a CytoFLEX flow cytometer (Beckman Coulter, Brea, CA, USA), collecting the entire cell suspension to quantify the proportion of CD31-positive cells relative to total events. Experiments were performed in triplicate. Data were analyzed using CytExpert software (version 2.4, Beckman Coulter, Brea, CA, USA) and FlowJo software (version10, BD Biosciences, Ashland, OR, USA).

### 2.10. Proliferation Assay

ECFCs and MSCs were seeded at 50,000 cells per well in 24-well plates containing PLA discs from the selected optimal condition 2 (impregnation after printing, 35 °C, 400 bar) and subsequently discs from condition 1 (impregnation after printing, 35 °C, 100 bar). Cultures were maintained in 500 µL of basal EGM-2 medium for 72 h at 37 °C in a humidified atmosphere with 5% CO_2_. All experiments were performed in triplicate.

After 72 h, cells were washed with 1× PBS (Gibco, Thermo Fisher Scientific, Waltham, MA, USA), fixed with 4% paraformaldehyde, and permeabilized with 0.2% Triton X-100 (Sigma-Aldrich, St. Louis, MO, USA) in 1× PBS (0.2% PBT) for 30 min under gentle agitation. Samples were blocked with 2.5% bovine serum albumin (BSA) (Sigma-Aldrich, St. Louis, MO, USA) in 0.2% PBT and incubated overnight at 4 °C with rabbit anti-Ki67 primary antibody (PA5-16785; Invitrogen, Thermo Fisher Scientific, Waltham, MA, USA) diluted 1:500 in 2.5% BSA. Samples were then incubated for 1 h at room temperature in the dark with Alexa Fluor 488-conjugated anti-rabbit secondary antibody (A-11008; Thermo Fisher Scientific, Waltham, MA, USA) diluted 1:1000, followed by nuclear counterstaining with 4′,6-diamidino-2-phenylindole (DAPI) (Sigma-Aldrich, St. Louis, MO, USA) diluted 1:5000 in 2.5% BSA.

Images were acquired at 10× magnification using an Olympus IX81 fluorescence microscope (Olympus Corporation, Tokyo, Japan). Quantification was performed using ImageJ software. DAPI staining was used to determine the total number of nuclei, and Ki67 positivity identified proliferating cells. Proliferation is expressed as the percentage of Ki67-positive nuclei relative to total DAPI-positive nuclei for each experimental condition.

### 2.11. Apoptosis Assay

To assess whether incorporation of mango leaf extract into PLA discs modulated apoptosis in ECFCs and MSCs, flow cytometry was performed using Annexin V (Cat. 560506; BD Biosciences, San Jose, CA, USA) and propidium iodide (Cat. 556463; BD Biosciences, San Jose, CA, USA). Annexin V and propidium iodide staining enabled discrimination of viable cells (Annexin V-negative, propidium iodide-negative), early apoptotic cells (Annexin V-positive, propidium iodide-negative), and late apoptotic or necrotic cells (Annexin V-positive, propidium iodide-positive).

Apoptotic status was evaluated after maintaining the cultures for 72 h under the previously described experimental setup. At the end of this period, the cells were recovered and subjected to Annexin V/propidium iodide staining in accordance with the supplier’s protocol. The stained suspensions were then examined using a CytoFLEX flow cytometer (Beckman Coulter, Brea, CA, USA). Three independent assays were carried out for each condition, and the cytometry files were interpreted with FlowJo software (BD Life Sciences, Ashland, OR, USA).

### 2.12. Statistical Analysis

Statgraphics Centurion 18 software was used to analyze extract loading in impregnated PLA. Analysis of variance was performed, and results are presented using a Pareto chart to identify the most influential variables affecting the impregnation process.

Statistical analyses related to the biological experiments were carried out with GraphPad Prism 9. Data distribution was first examined with the Shapiro–Wilk test, whereas equality of variances was checked using the Brown–Forsythe test. When the assumptions for parametric analysis were satisfied, differences among groups were examined by one-way ANOVA, followed by Tukey’s multiple-comparison procedure. In contrast, non-parametric datasets were analyzed with the Kruskal–Wallis test, and pairwise differences were explored with the Mann–Whitney U test when required. Statistical significance was established at *p* < 0.05 using two-sided testing.

## 3. Results and Discussion

### 3.1. Characterization of Mango Leaf Extract

Enhanced solvent extraction (ESE) was explored in this work as a technique to obtain MLE with high antioxidant content. Although bioactive compounds have traditionally been recovered from plants using conventional extraction methods, the increasing demand for safety in the food, cosmetic, biomedical, and pharmaceutical industries, driven by consumer health and environmental protection, has prompted the search for more efficient and sustainable processes. In this context, this work examined an eco-friendly extraction technique based on high-pressure technologies. Increasing the pressure enhances the contact surface area between the matrix and the solvent, allowing the solvent to penetrate areas of the solid matrix that would not normally be accessible at ambient pressure. Furthermore, increasing the extraction temperature promotes the release of analytes from the solid matrix and their diffusion into the solvent, resulting in higher partition coefficient values [38]. Another advantage of ESE is the use of high proportions of CO_2_, which reduces the consumption of liquid solvents. However, the presence of ethanol is essential to recover high-to-medium polarity bioactive compounds, such as mangiferin, quercetin, and gallic acid [39].

A maximum global extraction yield of 10.83 ± 0.91% (*w*/*w*, dried leaves) was obtained using CO_2_/ethanol (1:1 *v*/*v*) at 200 bar and 80 °C (Table 1). According to the literature [36], plant extracts show poor antioxidant activity when AAI < 0.5, moderate activity between 0.5 and 1.0, strong activity between 1.0 and 2.0, and very strong activity when AAI > 2.0. The obtained extract presented an AAI value of 2.36 ± 0.01, corresponding to a very strong antioxidant capacity. This high activity is in agreement with the high total polyphenol content, which includes major phenolic compounds such as mangiferin, iriflophenones, and gallic acid (Table 1).

A previous study [34] evaluated the same extraction technique under the same operating conditions, obtaining a similar extraction yield of 10.7%, with lower AAI (AAI = 0.7703) than reported here. The AAI presented in this study is considerably higher than that reported in the previous publication. This confirms that the MLE obtained has a potent antioxidant capacity that could be beneficial in biomedicine. It is known that the extraction method and the climatic conditions in which the plant grows both have an important influence on the extraction yield [40], and it seems that it is the latter factor that has caused the increase in antioxidant activity in the extract obtained.

### 3.2. Supercritical Impregnation

The Supercritical Solvent Impregnation (SSI) process typically follows three distinct stages: (1) dissolution of the target compounds in scCO_2_; (2) saturation of the solid matrix with the solutes; and (3) system depressurization for scCO_2_ removal. In the first stage, scCO_2_ serves as the solvent for the target compounds. Simultaneously, it diffuses into the carrier material, inducing swelling and plasticization, which enhances mass transfer. The solubility of the solutes in the supercritical phase is governed by their chemical nature, as well as the operational pressure and temperature. During the second stage, the equilibrium between scCO_2_, the solutes, and the solid matrix is critical for successful adsorption. For effective impregnation, the affinity between the target compounds and the solid matrix must surpass their affinity for the supercritical solvent. This favorable interaction ensures the migration of the solutes from the supercritical phase onto the adsorbent’s surface or into its pores. This phase also depends on the pressure and temperature conditions used. In the final stage, the system is depressurized, causing CO_2_ to revert to a gas, which is then removed from the system and potentially recycled. As a result, the target compounds can be impregnated on the solid matrix [38].

**Figure 2:**
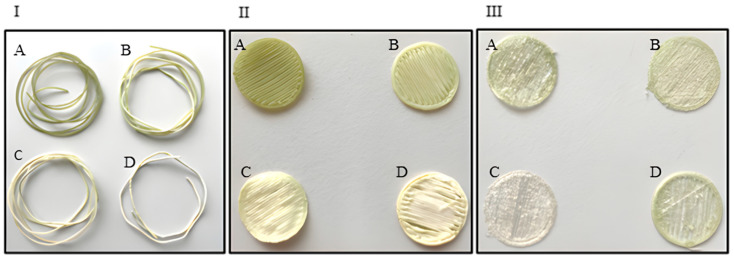
(**I**) Impregnated PLA filaments; (**II**) 3D-printed discs + SCI; and (**III**) SCI + 3D-printed discs impregnated under the following conditions: (**A**) T = 35 °C, *p* = 100 bar; (**B**) T = 35 °C, *p* = 400 bar; (**C**) T = 55 °C, *p* = 100 bar; and (**D**) T = 55 °C, *p* = 400 bar.

Figure 2 shows the results obtained by operating at different pressure and temperature values. Two distinct processing sequences were evaluated: (i) filament impregnation followed by 3D printing (SCI + 3D, Figure 2I,III) and (ii) post-printing impregnation of the discs (3D + SCI, Figure 2II). The core objective of comparing these methodologies and their respective operating conditions is to determine their subsequent impact on cell culture performance.

Comparing Figure 2II,III shows that the discs in the latter are more deformed under all impregnation conditions than those in Figure 2II. During impregnation, the polymer’s free volume increases due to its plasticization, a process known as swelling. This can result in variations in the diameter and length of the filament. Since the filament is fed into the printer, the swelling effect may render it unsuitable for printing purposes. The results obtained agree with those previously observed [29,33,34] and are important in avoiding an excessive increase in the polymer’s diameter, as this causes impediments in the printing process.

**Figure 3:**
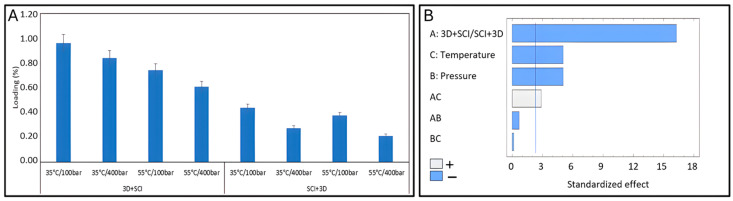
(**A**) MLE loading under different impregnation conditions (*n* = 2). Values are presented as mean ± standard error of the mean (SEM). (**B**) Pareto chart showing MLE loading.

Figure 3A shows MEL loading (%) for the different operating conditions studied. The values range from 0.61 to 0.97% for the 3D + SCI configuration, which is lower (from 0.21 to 0.41%) than for the other SCI + 3D configuration. Therefore, it can be inferred that the 3D + SCI configuration was preferable. In general, operating at 35 °C results in better impregnation performance than operating at 55 °C. An increase in temperature at constant pressure decreases the density of the scCO_2_, and therefore the affinity of the compounds for the supercritical phase decreases. This results in a low-scCO_2_/active compound solution, meaning less of the active compound can come into contact with the polymer; hence, the impregnation is lower. This effect is more pronounced in the 3D + SCI configuration than in the SCI + 3D configuration. On the other hand, the higher scCO_2_ density at higher pressures and constant temperature causes better affinity of the compounds to the supercritical phase, leading to lower impregnation values.

The Pareto diagram (Figure 3B) obtained by statistically processing the design of the experiment confirms that there is a high correlation between the MLE loading and the operating conditions. The most influential factor was the manner in which the process is carried out (3D + SCI/SCI + 3D). Pressure and temperature also have a negative effect, so working at 100 bar and 35 °C is advisable to obtain the highest loads. The interaction between temperature and pressure has been shown to significantly affect MLE loadings, probably due to the correlation between supercritical carbon dioxide (scCO_2_) density and these two variables.

**Figure 4:**
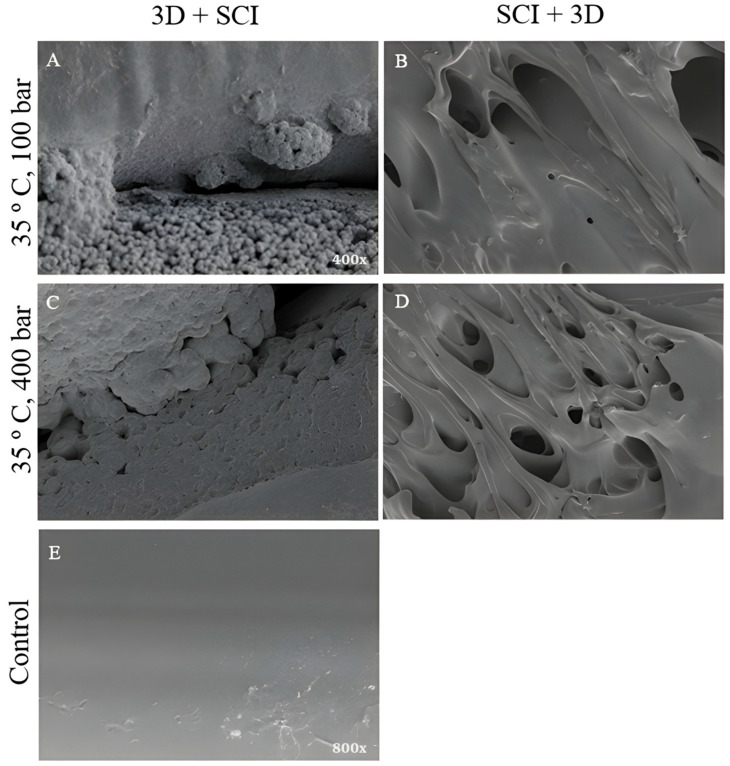
Scanning electron microscopy of the samples: (**A**) 3D + SCI, T = 35 °C, *p* = 100 bar (400×), (**B**) SCI + 3D, T = 35 °C, *p* = 100 bar (400×), (**C**) 3D + SCI, T = 35 °C, *p* = 400 bar (400×), (**D**) SCI + 3D, T = 35 °C, *p* = 400 bar (400×), (**E**) Unimpregnated PLA control disk (800×).

Microscopic examination was performed on filaments processed at 35 °C and 100–400 bar under the 3D + SCI and SCI + 3D configurations, together with non-impregnated PLA filaments used as controls, in order to identify surface changes associated with MLE incorporation. Representative images of these surface features are presented in Figure 4.

The unimpregnated PLA control discs (Figure 4E) mostly have a uniform and smooth surface. Hardly any irregularities were observed; those present may be intrinsic to the material. The 3D + SCI images (Figure 4A,C) differ significantly from the SCI + D images (Figure 4B,D). The 3D + SCI discs (Figure 4A,C) show the spherical shaped mango leaf extract coating the inside and outside of the polymer. Bumps are also visible in both images, probably due to particles lodged inside the polymer, as well as some surface pores resulting from the polymer swelling in the presence of the extract [33].

In the case of the SCI + 3D discs (see Figure 4B,D), deformation of the polymer conformation was observed, and no MLE-impregnated particles are visible. This may be because the filament is impregnated before being introduced into the printer, which increases its porosity and causes the diameter to increase by a few millimeters. This deformation is evident when the filament passes through the 3D printer. These images show that the internal structure of the PLA filament has changed. Such alterations could result in critical mechanical malfunctions that could affect the subsequent performance of the polymer when used to manufacture medical devices.

### 3.3. Cell Viability

**Figure 5:**
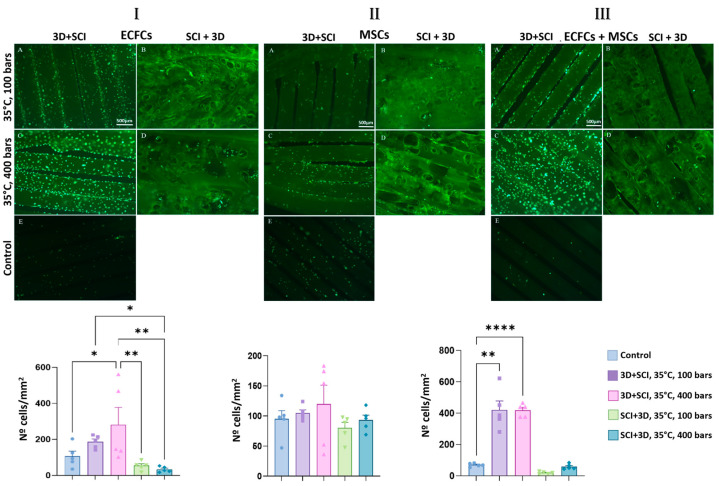
Viability of cultures on PLA discs impregnated with mango leaf extract under different conditions: (**I**) ECFCs, (**II**) MSCs, and (**III**) ECFCs + MSCs. (**A**) 3D + SCI, T = 35 °C, *p* = 100 bar; (**B**) SCI + 3D, T = 35 °C, *p* = 100 bar; (**C**) 3D + SCI, T = 35 °C, *p* = 400 bar; (**D**) SCI + 3D, T = 35 °C, *p* = 400 bar; (**E**) Control. Images were acquired using a fluorescence microscope with a 4x objective. Graphs show the number of cells counted per mm^2^ of PLA disc surface. Values are presented as mean ± SEM (*n* = 5). Asterisks indicate statistically significant differences: * *p* < 0.05, ** *p* < 0.01, and **** *p* < 0.0001.

Cell viability and adhesion on MLE functionalized PLA discs were assessed after 72 h of culture by Calcein staining and fluorescence microscopy. The quantitative outcomes obtained for each condition are shown in Figure 5I–III. A clear and consistent pattern emerged across ECFCs, MSCs and ECFC + MSC co-culture. The most prominent difference was the markedly higher number of adherent cells on discs manufactured by printing first and impregnating afterwards (3D + SCI) at 35 °C, compared with the remaining conditions. Within this 3D + SCI group, an additional pressure-dependent effect was observed, as discs impregnated at 400 bar supported a higher number of adherent cells than discs impregnated at 100 bar. This pressure-associated increase was consistently detected in wells seeded with ECFCs, in wells seeded with MSCs, and in ECFC + MSC co-culture. Importantly, all 3D + SCI conditions exhibited statistically significant differences relative to the unimpregnated PLA control discs in terms of adherent cell number (Figure 5I–III).

A notable change was observed when the polymer was impregnated prior to 3D printing (SCI + 3D). In this sequence, the impregnated filaments exhibited irregular and unstable surfaces that hindered disc fabrication. Consequently, the resulting SCI + 3D discs showed geometric deformation and loss of the original flat conformation, as illustrated in panels B and D of Figure 5I–III. Under these conditions, the presence of surface undulations led to non-uniform spatial distribution of adherent cells, which tended to concentrate in specific valley-like regions. This constrained colonization resulted in an overall reduction in the total number of cells adhering to the PLA discs [32].

In addition to the effects of material and processing, differences between cell types were apparent. MSC-seeded wells yielded lower adherent cell numbers than ECFC-seeded wells across all conditions. This may reflect intrinsic differences in adhesion behavior, as endothelial cells possess multiple adhesion-related proteins and readily attach to common culture substrates such as collagen, fibronectin and tissue culture-treated plastic, and can also attach, albeit less efficiently, to untreated glass [39]. Such inherent adhesion competence may become particularly relevant when surface geometry and topography are suboptimal, as in the SCI + 3D discs.

**Figure 6:**
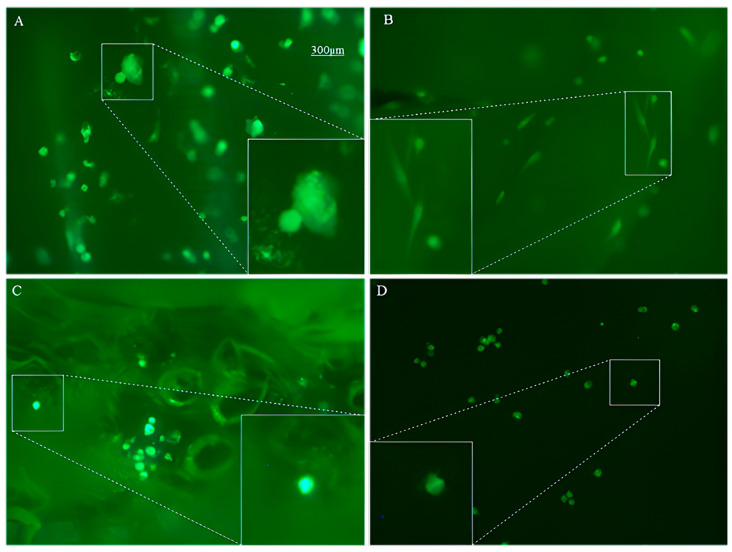
Analysis of cell morphology on PLA discs impregnated with mango leaf extract. Representative fluorescence microscopy images (10x objective) of ECFCs and MSCs cultured on PLA discs impregnated with MLE under different conditions, using an unimpregnated PLA disc as control: (**A**) ECFCs, T = 35 °C, *p* = 400 bar (3D + SCI); (**B**) MSCs, T = 35 °C, *p* = 400 bar (3D + SCI); (**C**) ECFCs + MSCs, T = 35 °C, *p* = 400 bar (SCI + 3D); (**D**) PLA control.

Calcein-based imaging not only enabled quantification of adherent cell density but also revealed condition-dependent differences in cell morphology and spatial organization. Representative 10X fluorescence micrographs are shown in Figure 6. Under the 3D + SCI workflow at 35 °C and 400 bar, ECFCs exhibited an extended, well-spread morphology consistent with the formation of a cohesive endothelial layer, whereas MSCs displayed a narrower, elongated spindle like shape, which is characteristic of healthy mesenchymal cells [41]. The observed morphology and spatial distribution suggest that MLE functionalization under these conditions provides a permissive microenvironment for cell attachment and maintenance, which may be linked to the bioactive properties of the extract, including the antioxidant activity reported above [42].

In contrast, for SCI + 3D discs (Figure 6C), where ECFCs + MSCs were seeded, the adherent cells did not exhibit the same spread morphology observed on 3D + SCI discs. Instead, their appearance was closer to that observed on unimpregnated control PLA discs (Figure 6D). Both conditions were characterized by a reduced number of adherent cells and a predominantly rounded, spherical morphology. Such contracted morphology is commonly associated with an unfavorable physiological state and has been linked to stress-induced cellular senescence, where cells lose their typical shape and adopt a more rounded phenotype when the microenvironment is not supportive for adhesion and proliferation [43].

Overall, the contrast between 3D + SCI and SCI + 3D was evident at the morphological level. Although SCI + 3D discs were also processed in the presence of MLE, the resulting cell morphology resembled that of the non-impregnated controls, suggesting that impregnation prior to printing may compromise either the effective presentation of bioactive cues or the integrity of the polymer surface environment after printing. A plausible explanation is that the printing step, which involves high temperatures, may reduce antioxidant activity associated with polyphenolic compounds [44] while simultaneously altering the internal structure of the polymer and its loading profile. In agreement with the adhesion quantification, MLE impregnation increased both the number of adherent cells and the overall culture quality when impregnation was performed after printing (3D + SCI).

### 3.4. Proliferation, Cell Type Identification, and Apoptosis Assays

**Figure 7:**
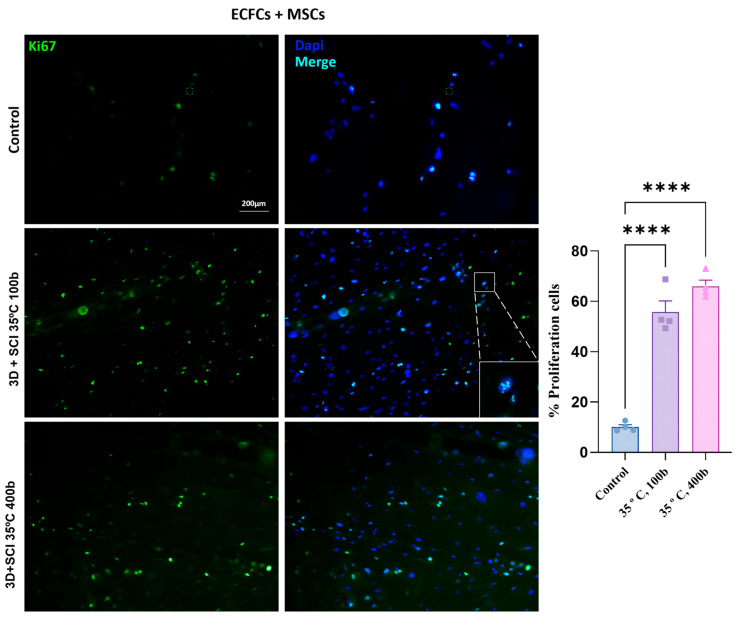
Effect of mango leaf extract on ECFC + MSC proliferation. Representative images of ECFC + MSC cultures on PLA discs impregnated with MLE under different conditions: 3D + SCI, T = 35 °C, *p* = 100 bar; 3D + SCI, T = 35 °C, *p* = 400 bar; Control. Images were acquired using a fluorescence microscope with a 10x objective. The graph shows the percentage of proliferating cells on PLA discs under the studied conditions. Values are presented as mean ± SEM (*n* = 4). *p*-value: **** < 0.0001.

Based on the results of the previous viability assay, condition 2 (3D + SCI, 35 °C, 400 bar) and condition 1 (3D + SCI, 35 °C, 100 bar) were selected as the optimal processing strategies. Consequently, they were used for immunofluorescence analysis of the ECFC + MSC coculture. The results are shown in Figure 7.

The proliferation percentages were 56.74% for condition 1 (3D + SCI, 35 °C, 100 bar), 66.01% for condition 2 (3D + SCI, 35 °C, 400 bar), and 10.74% for the control discs. Both impregnated conditions showed statistically significant differences relative to the control. These findings indicate that condition 2 (35 °C, 400 bar) promoted the highest proliferative fraction. This is consistent with previous assays identifying this temperature–pressure setting as the most favorable condition for ECFC and MSC viability, which also correlated with higher impregnated extract levels

In contrast to these results, several studies have reported anti-proliferative properties of MLE. This apparent discrepancy may be attributed to the low MLE concentration achieved in the current system (0.0096 mg/L), compared with concentrations evaluated in previous reports [32]. Therefore, it would be of interest to further explore the concentration threshold at which MLE begins to exert anti-proliferative effects, as this could enable the modulation of cell growth on the polymer surface, ensuring optimal coverage of the biodegradable material while maintaining controlled proliferation [45].

**Figure 8:**
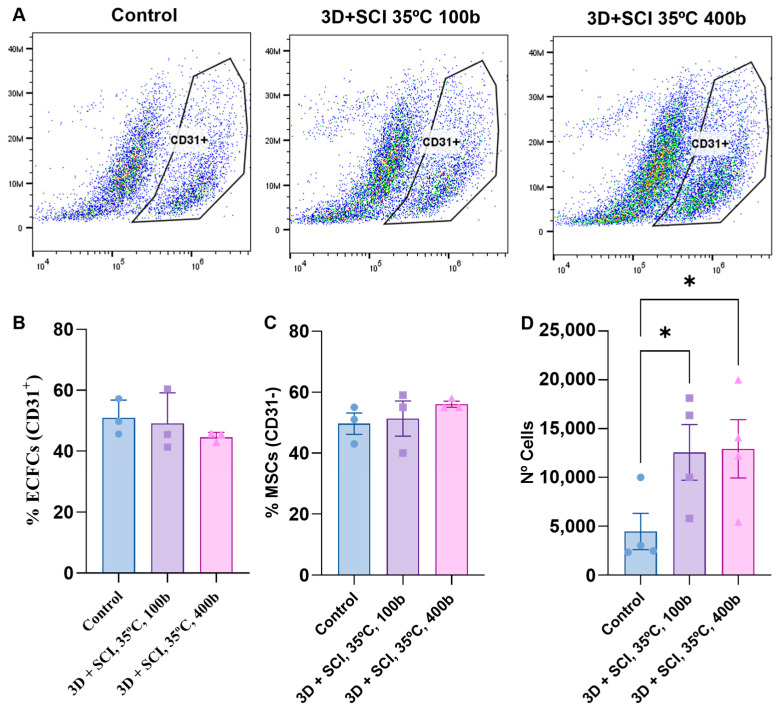
Effect of PLA discs impregnated with mango leaf extract on the proportion and number of adherent CD31-positive cells after 72 h of culture. (**A**) Representative flow cytometry dot plots showing the gated CD31+ cell population under the different experimental conditions: Control, 3D + SCI at 35 °C and 100 bar, and 3D + SCI at 35 °C and 400 bar. The polygonal line in each dot plot delineates the gating region used to identify CD31-positive cells. Dot colors reflect event density, with warmer colors indicating a higher concentration of events. (**B**) Percentage of ECFCs (CD31+). (**C**) Percentage of MSCs (CD31−). (**D**) Total number of cells adherent to PLA discs after 72 h of culture. Values are presented as mean ± SEM (*n* = 4). *p*-value: * < 0.05.

In addition, a flow cytometry-based assay was carried out to corroborate the presence of the two cell types and to determine whether there were significant differences in their adherence to PLA discs. Thus, following flow cytometric quantification, the percentage of CD31+ cells was representative of the endothelial fraction relative to total cells (Figure 8A). The CD31− percentage, corresponding to the mesenchymal fraction, and the total cell number were also determined, as summarized in Figure 8B–D.

This CD31-based identification approach provides additional information beyond relative lineage proportions, specifically the total cell count per sample. As shown in Figure 8D, significant differences were detected in the total number of adherent cells on MLE-impregnated PLA discs compared with unimpregnated controls, in agreement with the calcein-based viability assay. Figure 8B illustrates the percentage of CD31+ endothelial cells, which was similar across all conditions. Similarly, Figure 8C shows the percentage of CD31− mesenchymal cells, defined as the population lacking CD31 staining. Overall, the proportion of each cell type per well did not vary substantially relative to the control.

Taken together, these findings support the conclusion that MLE-impregnated PLA discs generated a more favorable environment for the maintenance and growth of both endothelial and mesenchymal cells [32] without apparent selectivity. In other words, within the conditions tested, MLE-impregnated PLA supported ECFCs and MSCs in comparable proportions, consistent with balanced co-culture maintenance.

**Figure 9:**
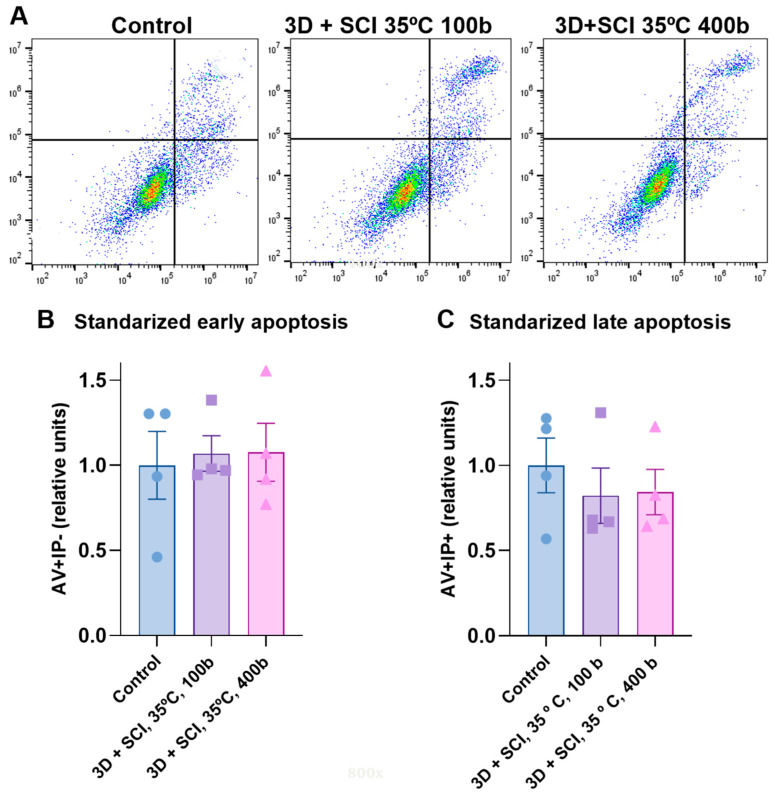
Effect of mango leaf extract on apoptosis of ECFCs + MSCs. (**A**) Representative dot plots showing apoptosis profiles of ECFCs + MSCs seeded on PLA discs under different conditions: (1) 3D + SCI, T = 35 °C, *p* = 100 bar; (2) 3D + SCI, T = 35 °C, *p* = 400 bar; (3) Control. (**B**,**C**) Graphs show the effect of MLE contained in PLA discs impregnated under different conditions on normalized early and late apoptosis. Values are presented as mean ± SEM (*n* = 4).

Finally, the influence of extract concentration on the apoptosis of ECFCs and MSCs was also evaluated by flow cytometry. No statistically significant differences were observed in early or late apoptosis between control and impregnated discs (Figure 9B,C). However, a trend toward reduced late apoptosis was observed in the impregnated discs, potentially linked to anti-apoptotic properties attributed to mango leaf extract in both cell types [32]. This trend may also suggest that the extract concentration was insufficient to elicit a clear anti-apoptotic effect [31], a conclusion that is conceptually consistent with the findings from the proliferation assay.

## 4. Conclusions

This study demonstrates that MLE can be successfully incorporated into PLA using scCO_2_-assisted impregnation, yielding a biofunctionalized and cytocompatible material. Enhanced solvent extraction produced an MLE fraction with high antioxidant capacity, supporting its suitability as a natural source of bioactive compounds for polymer functionalization [36,46,47].

A key outcome of this work is that the manufacturing sequence is the dominant determinant of both material fidelity and functional performance. The workflow based on 3D printing followed by impregnation (3D + SCI) preserved disc geometry and enabled higher MLE loading than impregnation prior to printing (SCI + 3D). The latter was associated with filament instability, disc deformation, and reduced effective cell colonization.

From a biological standpoint, MLE-impregnated PLA produced under the 3D + SCI workflow, particularly at 35 °C and 400 bar, supported increased adhesion and viability of ECFCs and MSCs, and enhanced the proliferative fraction in co-culture while maintaining comparable lineage proportions as assessed by CD31 flow cytometry. Furthermore, apoptosis analysis did not indicate pro-apoptotic effects under the tested conditions, supporting the material’s cytocompatibility.

Overall, these findings position post-printing supercritical impregnation as a robust, green and reproducible strategy to generate antioxidant PLA constructs from agro-industrial by-products. This approach holds significant potential for vascular-oriented tissue engineering and regenerative applications. Future studies should define the release kinetics and stability of the bioactive fraction, establish the concentration range at which MLE shifts toward anti-proliferative or anti-apoptotic effects, and expand functional endpoints and mechanical characterization to strengthen translational readiness [31,45].

## Figures and Tables

**Table 1:** Characterization of the extract.

Parameter	Value
Yield extraction (%)	10.83 ± 0.91
IC_50_ (µg/mL)	9.74 ± 0.03
AAI	2.36 ± 0.01
Phenolic compounds (g/100 g dried extract)	
gallic acid	2.57 ± 0.04
mangiferin	9.08 ± 0.02
iriflophenone 3-C-β-d-glucoside	11.12 ± 1.03
iriflophenone 3-C-(2-O-galloyl)-β-d-glucoside	2.72 ± 0.05
quercetin 3-d-galactoside	1.87 ± 0.08
quercetin 3-β-d-glucoside	1.52 ± 0.02

Results are expressed as mean value ± standard deviation (*n* = 2). AAI: Antioxidant Activity Index; IC50: concentration required to inhibit 50% of the DPPH radical signal.

## Data Availability

The original contributions presented in this study are included in the article. Further inquiries can be directed to the corresponding authors.

## Abbreviations

The following abbreviations are used in this manuscript:

AAI	Antioxidant activity index
AN V	Annexin V
CO_2_	Carbon dioxide
DAPI	4′,6 diamidino 2 phenylindole
DPPH	2,2 diphenyl 1 picrylhydrazyl
ECFCs	Endothelial colony-forming cells
EGM 2	Endothelial Growth Medium 2
FBS	Fetal bovine serum
GPS	Glutamine penicillin streptomycin
IC_50_	Half maximal inhibitory concentration
MLE	Mango leaf extract
MSCs	Mesenchymal stromal cells
PBS	Phosphate-buffered saline
PBT	PBS with Triton X 100
PI	Propidium iodide
PLA	Poly(lactic acid)
PFA	Paraformaldehyde
SCI	Supercritical impregnation
